# Clinical Benefits of Biochemical Markers of Fibrosis in Egyptian Children With Chronic Liver Diseases

**DOI:** 10.4021/gr246w

**Published:** 2010-11-20

**Authors:** Tawhida Y. Abdel-Ghaffar, Behairy E. Behairy, Azza Abd El-Shaheed, Karam Mahdy, Mohamed El-Batanony, Mohsen H. Hussein, Mostafa M. Sira

**Affiliations:** a Department of Pediatrics, Faculty of Medicine, Ain Shams University, Cairo, Egypt; b Department of Pediatric Hepatology, National Liver Institute, Menofiya University, 32511 Shebin El-koom, Menofiya, Egypt; c Department of Child Health, National Research Centre, El Bohouth Street, Dokki 12311, Cairo, Egypt; d Medical Biochemistry Department, National Research Centre, El Bohouth Street, Dokki 12311, Cairo, Egypt; e Department of Pathology, National Liver Institute, Menofiya University, 32511 Shebin El-koom, Menofiya, Egypt

**Keywords:** Liver fibrosis, Hepatitis C virus, Hepatitis B virus, Bilharziasis, Collagen IV, Liver fibrosis, Egyptian children

## Abstract

**Background:**

The need for repetition of liver biopsy, especially in assessing the degree of fibrosis and follow-up of treatment protocols, justifies an intensive search for non-invasive alternatives. We attempted to investigate the clinical usefulness of serum fibrogenesis markers in pediatric chronic liver diseases.

**Methods:**

We measured serum levels of TGF-β1, collagen IV, laminin, MMP-2 and EGF-R, in 50 children with chronic liver disease (HBV, HCV and Bilharziasis) and 30 healthy controls, and determined their relationship to frequently used liver function tests and liver biopsy findings in patients.

**Results:**

TGF-β1, collagen IV, laminin and MMP-2, but not EGF-R, were significantly higher in patients than in controls (P < 0.01). None of these markers correlated with the histological fibrosis stage, whereas laminin correlated with necroinflammatory activity (P < 0.01). TGF-β1, collagen IV, laminin and MMP-2 had the ability to discriminate patients with significant fibrosis, while only collagen IV and laminin were able to discriminate those with cirrhosis. Among these markers, collagen IV had the best predictive accuracy for significant fibrosis (AUROC 0.94; PPV 91.5%) and cirrhosis (AUROC 0.85; PPV 80%).

**Conclusions:**

In conclusion, these markers may be useful in reducing but not replacing the need for liver biopsy in the monitoring of disease progression and treatment effectiveness and might be an inseparable part of assessment of chronic hepatopathies.

## Introduction

Hepatic fibrosis is the final common path of liver injury in most chronic liver diseases and can lead to cirrhosis, which is responsible for the majority of clinical complications. Liver biopsy is not only essential in establishing the diagnosis, but also in assessing the degree of fibrosis. Although liver biopsy has long been considered to be the gold standard of fibrosis assessment, the procedure is invasive, potentially associated with complications, and provides only a semi-quantitative assessment. Its repetition is required for following up the disease progression and monitoring treatment efficacy. This repetition is not easily accepted by patients especially in the pediatric population. Moreover, the diagnostic accuracy of liver biopsy in the staging of fibrosis is seriously affected by errors in sampling and inter-observer variation [1, 2]. These drawbacks justify an intensive search for non-invasive alternatives that are safe, inexpensive and reliable [3]. Some serum markers have been described to correlate with liver fibrosis [4]. Non-invasive diagnosis of liver fibrosis has been extensively evaluated in adult populations [5-7]. In contrast, in pediatric population, data are lacking and liver biopsy is still the only reliable tool for diagnosing the histological features [8].

Fibrosis is characterized by excess deposition of extra-cellular matrix (ECM) components including different collagens and non-collagenous proteins such as laminin, fibronectin, undulin, and so on [9]. The key cellular mediator of fibrosis is the hepatic stellate cells (HSCs) which when activated serve as the primary collagen-producing cell. HSCs are activated by a variety of mechanisms, including cytokines, chemokines and others [10]. Activated HSCs release transforming growth factor-beta1 (TGF-β1). This cytokine highly stimulates fibrogenesis by HSCs [11]. At the site of hepatic injury, damaged platelets release platelet derived growth factor and epidermal growth factor (EFG) which are strong stimulants for proliferation of HSCs [12]. There is continuous ECM deposition and degradation at the same time. Degradation is mediated by enzymes called matrix metalloproteinases (MMPs). In hepatic fibrosis, there is an increase in MMP-2 (collagenase IV or gelatinase A) leading to increased collagen IV destruction. Fibrosis mediators, enzymes and ECM breakdown products enter the circulation and thus potentially reflect fibrogenesis or fibrolysis [13].

The aim of this study was to evaluate the clinical benefits of serum markers of fibrosis in children with chronic liver disease (chronic viral hepatitis and Bilharziasis).

## Subjects and Methods

### Study population

Eighty children were enrolled in this study, 50 patients with chronic liver disease recruited from the National Liver Institute, Menofiya University, outpatient clinic and inpatient ward, and 30 apparently healthy children with no history or clinical evidence of liver disease or any other disease, attending the outpatient clinic for routine check-up, who served as controls. They were divided into four groups: chronic hepatitis B virus (HBV) group (12 patients), chronic hepatitis C virus (HCV) group (29 patients), Bilharziasis group (9 patients) and control group (30 children). A signed informed consent was obtained from the parents of all the patients and controls before enrollment in the study. The study was approved by the Research Ethics Committee of National Liver Institute, Menofiya University.

### Etiological diagnosis

HBV infection was defined by positive hepatitis B surface antigen, hepatitis B core IgM or hepatitis B core IgG with liver biopsy features of HBV infection. HCV infection was defined by positive anti-HCV and detection of HCV-RNA by qualitative polymerase chain reaction (PCR). Bilharziasis was defined by positive rectal snip for bilharzial ova (8 patients) or passage of bilharzial ova in stool (1 patient) together with the presence of periportal fibrosis on liver ultrasound and absence of viral markers.

### Sample collection

Blood samples were collected under complete aseptic technique from both patients and controls. Blood was allowed to clot naturally in the test tube, serum was then separated by centrifugation, divided into small aliquots and stored immediately at -80°C till time of use. EDTA was added to a second sample for complete blood count (CBC) and was tested immediately. Na citrate was added to a third sample for assessing prothrombin concentration (PC).

### Liver biopsy

Ultrasonography-guided liver biopsy was done for chronic hepatitis patients only. Liver biopsies were performed using true cut needle. Biopsy specimens were fixed in formalin and embedded in paraffin. Liver fibrosis and necroinflammatory activity were evaluated according to Ishak staging and grading score where histological activity index (HAI) ranged from 0 to 12 while fibrosis score ranged from F0 to F6 [14].

### Serum biochemical markers assay

One aliquot was thawed and all individuals, patients and controls, were tested for liver function tests (LFTs) including total and direct bilirubin (TB and DB), aspartate transaminase (AST), alanine transaminase (ALT), alkaline phosphatase, gamma glutamyl transferase (GGT), total proteins, and serum albumin. The other aliquots were thawed and assayed for TGF-β1, collagen IV, MMP-2, laminin and epidermal growth factor receptor (EGF-R). TGF-β1 was measured by ELISA (DRG International Inc., USA) according to the method described by Kropf et al [15]. Collagen IV was measured by ELISA (Biotrin International LTD, Ireland) as described in Tsutsumi et al [16]. Total MMP-2 was measured using Quantikine immunoassay kit (R&D systems, USA) according to the method of Murphy [17]. Laminin was measured by enzyme immunoassay kit (Takara, Japan) according to the method described by Burgeson et al [18]. EGF-R assayed by ELISA (Bender MedSystems, Austria) as described in Gamou and Schimizu [19].

### Statistical tools

Descriptive results were expressed as mean ± standard error of mean (mean ± SEM) or number (percentage) of individuals with a condition. Statistical significance between multiple groups was tested using non-parametric Kruskal-Wallis test. Significance between individual groups was tested either by nonparametric Mann-Whitney U test or Pearson's χ^2^ test. Correlations were tested by Spearman's correlation. The diagnostic value of serum biochemical markers was assessed by calculating the area under the receiver operator characteristic (ROC) curves. The diagnostic performance of the non-invasive markers for significant fibrosis and cirrhosis was measured as sensitivity, specificity, positive predictive value (PPV) and negative predictive value (NPV). Sensitivity, specificity, PPV and NPV were expressed as percentage. The cut-off values for optimal clinical performance were determined from the ROC curves. Results were considered significant if P value was no more than 0.05 (**P ≤ 0.01; *P ≤ 0.05). Statistical analysis was performed using SPSS software v.13.

## Results

### Study individuals' characteristics

Demographic, clinical and laboratory characteristics of the 80 studied individuals were described in Table 1. The patients and controls were age and sex matched (P > 0.05 for both) and there was a statistical significant difference between the studied groups as regards TB, ALT, AST and Alk. Ph, while there were no significant differences as regards the other parameters.

**Table 1:** Demographic, Clinical and Laboratory Characteristics of the Studied Patients

Characteristics		HBV (n = 12)	HCV (n = 29)	Bilharziasis (n = 9)	P-value
Age (years)	Patients	10.75 ± 1.04	11.54 ± 0.63	11.89 ± 1.1	0.206
	Controls 10.5 ± 0.46				
Male n (%)	Patients	10 (83.3%)	22 (75.9%)	9 (100%)	0.355
	Controls 22 (73.3%)				
Hematemesis n (%)		1 (8.3%)	4 (13.8%)	3 (33.3%)	0.267
Epistaxis n (%)		1 (8.3%)	4 (13.8%)	2 (22.2%)	0.662
Hepatomegaly n (%)		2 (16.6%)	8 (27.6%)	2 (22.2%)	0.751
Splenomegaly n (%)		7 (58.3%)	19 (56.5%)	8 (88.8%)	0.301
Ascites n (%)		2 (16.6%)	4 (13.8%)	3 (33.3%)	0.506
Hb (g/dl)		10.5 ± 0.42	10.89 ± 0.34	11.18 ± 0.39	0.659
WBCs (x 10^3^/mm^3^)		6.38 ± 0.95	7.2 ± 0.49	7.04 ± 0.67	0.624
Platelets (x 10^3^/mm^3^)		135.4 ± 17.3	161.9 ± 14.1	196.7 ± 17.03	0.64
TB (mg/dl)		1.83 ± 0.22	1.32 ± 0.14	0.95 ± 0.16	0.026*
ALT (U/L)		72.58 ± 12.41	107.37 ± 28.1	30.67 ± 3.21	0.03*
AST (U/L)		71.17 ± 10.76	120.07 ± 21.78	38.56 ± 6.09	0.002**
Albumin (g/dl)		3.49 ± 0.19	3.57 ± 0.11	3.8 ± 0.1	0.35
Alkaline phosphatase (U/L)		214.33 ± 41.18	165.19 ± 28.86	80.33 ± 12.42	0.02*
GGT (U/L)		54.33 ± 15.98	59.87 ± 11.06	38.78 ± 9.64	0.61
PC (%)		69.42 ± 6.03	65.61 ± 2.46	75.67 ± 4.1	0.163

Quantitative data are expressed as mean ± SEM and qualitative data as number and %. Significance was tested using non-parametric Kruskal-Wallis test or Chi-square test. *P ≤ 0.05, **P ≤ 0.01

### Serum levels of fibrosis markers in the studied groups

**Figure 1:**
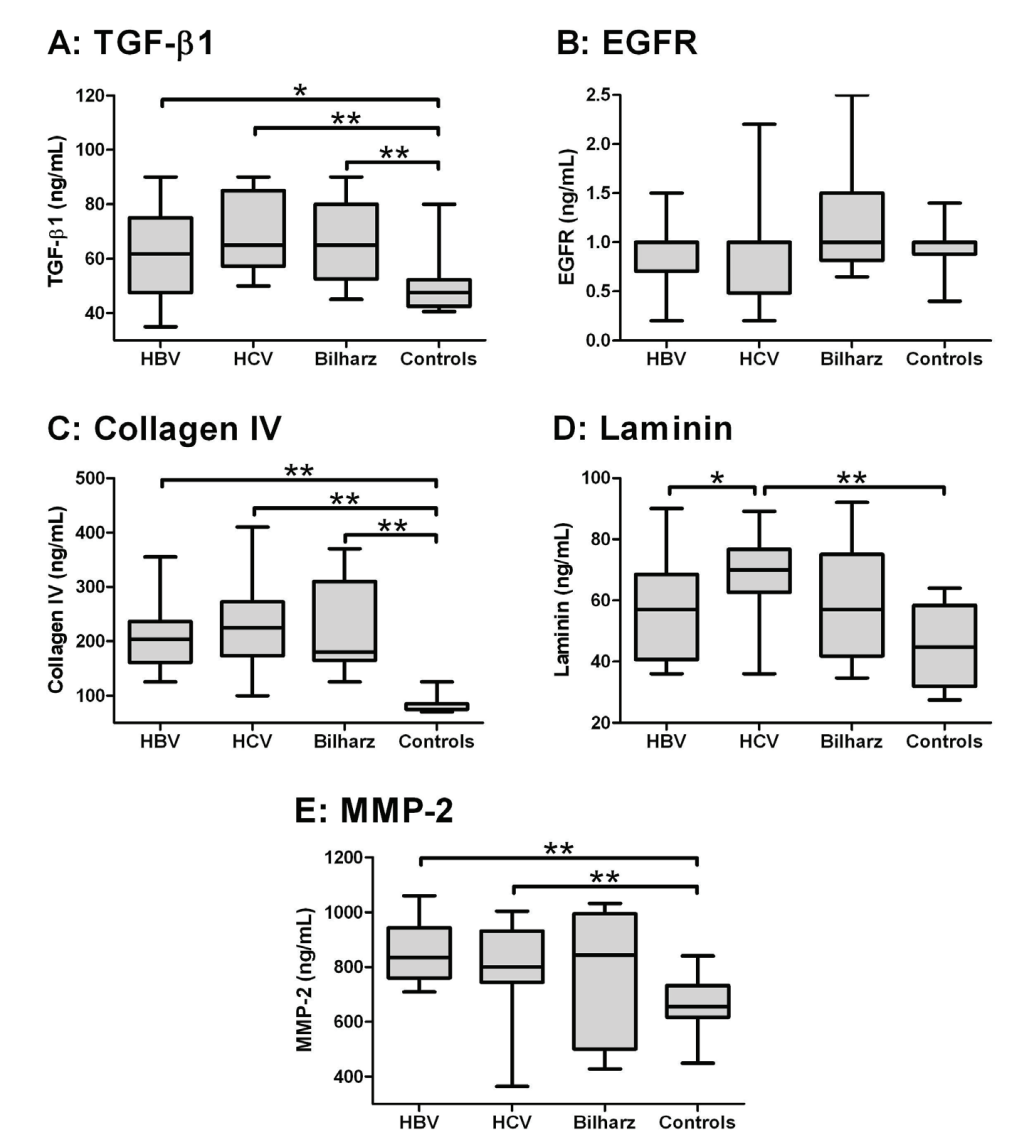
Box-and-whiskers plot for serum biochemical markers. The top and bottom of each box are the 25th and 75th centiles. The line through the box is the median and the error bars are the maximum and minimum. The horizontal bar represents the significance between the designated groups. * P ≤ 0.05 and ** P ≤ 0.01.

Using Kruskal-Wallis test, there was a statistical significant difference among the four studied groups as regards TGF-β1, collagen IV, laminin and MMP-2 (P < 0.000), while there was no significant difference as regards EGF-R (Table 2). When comparing the studied groups individually with each other (Fig. 1), TGF-β and collagen IV were significantly higher in HBV, HCV and bilharzial group than in the control group with no significant difference between the three patients groups. Laminin in the HCV group was significantly higher than in the HBV and the control groups but not the Bilharziasis group; similarly, MMP-2 was significantly higher in HBV and HCV groups than in the controls but not in the Bilharziasis group. On the other hand, there was no significant difference between any of the studied groups as regards EGFR, yet it was still higher in the Bilharziasis group than in the HBV, HCV and control groups.

**Table 2:** Serum Levels of Biochemical Markers in the Studied Groups

Marker	HBV n = 12	HCV n = 29	Bilharziasis n = 9	All Patients n = 50	Controls n = 30	P-value^a^	P-value^b^
TGF-β1 (ng/ml)	61.7 ± 4.9	68.7 ± 2.6	65.5 ± 5.29	66.504 ± 2.14	50.9 ± 2.2	0.000**	0.000**
EGF-R (ng/ml)	0.904 ± 0.1	0.929 ± 0.1	1.2 ± 0. 19	0.97 ± 0.07	0.88 ± 0.05	0.315	0.699
Collagen IV (ng/ml)	211.1 ± 19.4	224.9 ± 14.3	226.1 ± 29.5	221.8 ± 10.7	85.6 ± 3.6	0.000**	0.000**
Laminin (ng/ml)	57.0 ± 4.8	68.9 ± 2.5	57.5 ± 6.6	64.05 ± 2.3	45.5 ± 2.4	0.000**	0.000**
MMP-2 (ng/ml)	849.3 ± 31.3	787.6 ± 33.6	762.2 ± 79.8	797.87 ± 25.18	653.4 ± 18.3	0.000**	0.000**

a Significance was tested between all patients (n = 50) versus controls (n = 30) using Mann-Whitney test.

b Significance was tested among individual etiological (HBV, HCV and Bilharziasis) groups and controls using Kruskal-Wallis test.

** P < 0.01. Values are expressed as mean ± standard error of mean (SEM).

### Correlation of fibrosis biochemical markers with CBC, LFTs in all the patients and with histopathological scores in chronic hepatitis B and C patients

Serum level of the five biochemical markers did not correlate with each other or with any of the LFTs or CBC components, except for TGF-β1 which showed a significant positive correlation (P < 0.05) with platelet count. Only 19 patients with chronic viral hepatitis had liver biopsy, but none of the bilharzial patients. Serum levels of the tested biochemical markers did not correlate with the stage of fibrosis or necroinflammatory activity, except for laminin which had significant direct correlation with necroinflammatory activity (P < 0.01) (Table 3).

**Table 3:** Correlation Analysis of Biochemical Markers With Age and Laboratory Parameters in All the Patients (n = 50) and With HAI and Stage of Fibrosis in Chronic Hepatitis B and C Patients Only (n = 19)

Variable			TGF-β1	EGFR	Collage IV	Laminin	MMP-2
EGFR		r	-0.25				
		P	0.079				
Collage IV		r	0.126	-0.156			
		P	0.382	0.28			
Laminin		r	0.024	0.034	0.205		
		P	0.867	0.817	0.153		
MMP-2		r	0.116	-0.030	-0.018	0.048	
		P	0.423	0.838	0.899	0.739	
Age	r		0.02	-0.122	0.202	0.044	0.103
	P		0.892	0.397	0.159	0.761	0.475
PLT	r		0.29	0.147	0.081	0.196	0.159
	P		0.041*	0.308	0.574	0.172	0.27
TBIL	r		-0.063	0.109	-0.053	0.093	0.068
	P		0.663	0.451	0.716	0.52	0.637
ALT	r		-0.027	-0.194	0.056	0.187	0.021
	P		0.854	0.178	0.701	0.193	0.886
AST	r		-0.083	-0.172	0.014	0.186	-0.125
	P		0.568	0.232	0.924	0.196	0.386
Albumin	r		0.155	0.017	0.099	-0.058	0.073
	P		0.283	0.906	0.495	0.687	0.616
ALP	r		0.047	-0.213	-0.066	0.033	0.236
	P		0.746	0.137	0.648	0.817	0.099
GGT	r		-0.06	0.011	0.123	0.104	0.075
	P		0.68	0.941	0.395	0.474	0.607
P.C	r		0.045	0.103	0.042	-0.016	0.11
	P		0.757	0.476	0.774	0.912	0.448
HAI	r		-0.226	0.117	0.138	0.636	0.072
	P		0.351	0.635	0.573	0.003**	0.769
Fibrosis stage	r		-0.196	0.005	-0.026	-0.083	-0.307
	P		0.421	0.985	0.915	0.734	0.201

r = correlation coefficient, *P ≤ 0.05, **P ≤ 0.01

### Diagnostic performance of fibrosis biochemical markers for discriminating significant fibrosis and cirrhosis

**Figure 2:**
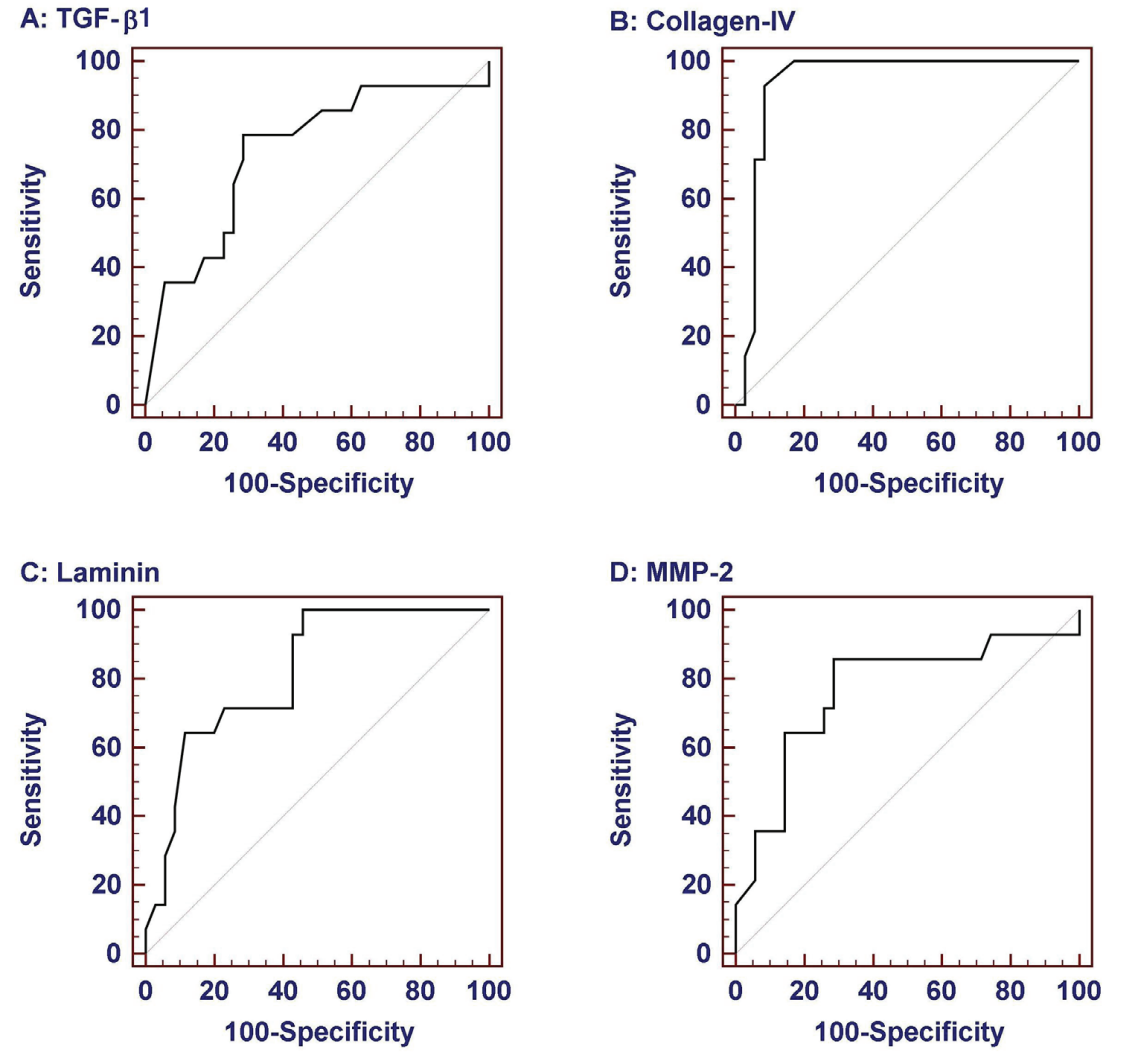
Receiver-operator characteristic (ROC) curves of serum biochemical markers for prediction of significant fibrosis (Ishak score > 2). Data were analyzed considering patients with liver biopsy in the chronic hepatitis group. Among them collagen IV was the most sensitive and specific.

Considering that significant fibrosis equals Ishak score more than 2 and cirrhosis equals Ishak score 6 [20], we estimated the area under the ROC curve (AUROC), the sensitivity, specificity, PPV, NPV and cut off values identifying individuals with significant fibrosis and those with cirrhosis (Fig. 2 and Table 4). TGF-β1 more than 54.8 ng/ml had a sensitivity of 78.6% and specificity of 71.4% in identifying significant fibrosis (Fig. 2A), and TGF-β1 more than 57.5 ng/ml had a sensitivity of 85.7% and specificity of 66.6% in identifying cirrhosis. Collagen IV at a cut off value of no less than 132.5 ng/ml had a sensitivity of 92.9% and specificity of 91.4% identifying significant fibrosis (Fig. 2B) and at a cut off value of more than 147.5 ng/ml had a sensitivity of 85.7% and specificity of 78.6% in identifying cirrhosis. Laminin at a cut off value of more than 60.9 ng/ml had a sensitivity of 71.4% and specificity of 77.1% in identifying significant fibrosis (Fig. 2C) and at a cut off value of more than 63.7 ng/ml had a sensitivity of 71.4% and specificity of 81% in identifying cirrhosis. MMP-2 at a cut off value of more than 742 ng/ml has a sensitivity of 85.7% and specificity of 71.4% in identifying significant fibrosis (Fig. 2D) and at a cut off value of more than 747 ng/ml had a sensitivity of 85.7% and specificity of 64.6% in identifying cirrhosis. Using different combinations of TGF-β1, Collagen, laminin and MMP2 did not significantly increase the performance over the use of each marker alone.

**Table 4:** Diagnostic Performance of Serum Biochemical Markers in Predicting Significant Fibrosis and Cirrhosis in Chronic Hepatitis B and C Patients

Marker	AUROC	P-value	Cut-off	Sensitivity	Specificity	PPV	NPV
Significant Fibrosis (Ishak score > 2)							
TGF-β1	0.74	0.01**	54.8	78.6	71.4	73.3	76.9
Collagen IV	0.94	0.000**	132.5	92.9	91.4	91.5	92.8
Laminin	0.82	0.001**	60.9	71.4	77.1	75.7	72.9
MMP-2	0.77	0.004**	742	85.7	71.4	74.9	83.3
Cirrhosis (Ishak score 6)							
TGF-β1	0.7	0.089	57.5	85.7	66.6	72	82.3
Collagen IV	0.85	0.003**	147.5	85.7	78.6	80	85.1
Laminin	0.76	0.031*	63.7	71.4	81	78.9	73.9
MMP-2	0.68	0.126	747	85.7	64.3	70	81.8

*P ≤ 0.05, **P ≤ 0.01

## Discussion

The aim of the present study was to evaluate the clinical benefits of serum biomarkers of liver fibrosis in pediatric chronic liver diseases. For this purpose, we employed five serum biological markers (TGF-β1, collagen IV, MMP-2, laminin and EGF-R) which are assumed to reflect the degree of liver fibrosis and compared these markers in 50 pediatric patients with chronic liver diseases to 30 healthy controls. Of these markers, TGF-β1, collagen IV, laminin and MMP-2 were significantly higher in diseased than in healthy controls. Comparing individual etiologic groups to controls, biochemical markers had the same significance in HCV and HBV patients. This significant elevation might reflect the fibrogenic process in the liver.

In the normal liver, sinusoidal endothelial cells and Kupffer cells have relatively high constitutive levels of TGF-β1, whereas HSCs express very little TGF-β1 in the normal state, and hepatocytes essentially none. When injury strikes, inflammatory cells are drawn to the site of injury and HSCs undergo activation, becoming myofibroblasts, which migrate, proliferate and become fibrogenic and contractile. This is due to TGF-β1 upregulation, increased activation, and increased receptor expression and signaling components [21, 22]. Luo et al found a significant elevation of TGF-β1 in liver cirrhosis, yet its correlation with activity was moderate [23]. In our study, TGF-β1 did not correlate with disease activity or with stage of liver fibrosis. In accordance with our results, Yang et al reported that TGF-β1 did not correlate to the severity of liver disease [24]. On the other hand, Zhang et al found that the level of TGF-β1 in chronic liver disease was comparable to that of the control group [25]. Our results showed that TGF-β1 significantly correlated with platelet count. This finding is explained by the fact that platelets are a major source of TGF-β1; moreover, platelets play a role in activation of its latent form [26].

Although it was reported that the expression of EGF and its receptor increase in cirrhosis and in viral liver disease [12, 27], we found that levels of EGF-R were comparable in HBV, HCV, bilharziasis and the controls. Yet, it was still higher in the bilharziasis group than in the other three studied groups. Abdel Rahman et al reported that EGF, which is the ligand for EGF-R, was indirectly stimulated by the bilharzial infection [27]. This may lead to EGF-R upregulation. Another possibility is that *Schistosoma mansoni* gene encodes a homologue of human EGF-R. This gene sequence has been characterized and found to encode a protein which shares substantial sequence and structural homology with other members of the EGF-R family [28, 29]. In our study, bilharzial patients were of mansoni type. This would explain the higher level of EGF-R in this group compared to HBV, HCV and control groups.

Similar to our results, Friedman et al and others [11, 30, 31] found that serum levels of collagen IV and laminin were significantly higher in patients with hepatic disorders than in healthy controls. Although in our study, laminin was found to be the only marker that highly correlates with HAI (P < 0.01) in the viral group, Lu et al [32] reported that laminin has no diagnostic value either in inflammation or in fibrotic changes in patients with chronic liver disease.

In agreement with our results, Kasahara et al and Walsh et al found that serum levels of MMP-2 were elevated in patients than in controls [33, 34]. On the other hand, Boeker et al [35] and Mueawaki et al [36] reported that serum MMP-2 concentrations were not altered in chronic liver disease. Taking the etiology into consideration, we could demonstrate that patients with viral etiology had significantly higher serum MMP-2 levels than the controls, whereas in patients with bilharziasis, the serum level of MMP-2 was comparable to that of the controls.

Paduch and Kandefer-Szerszen [37] demonstrated the inhibitory effect of vitamin D on MMP2. Furthermore, the addition of vitamin D to neoplastic cell cultures suppressed MMP2 secretion. Timms et al [38] have shown vitamin D deficiency to be associated with significant increase in MMP2. Arteh et al [39] and Goerge et al [40] reported vitamin D deficiency in chronic hepatitis B and C where lower levels were found in patients with liver cirrhosis than in non-cirrhotics. Moreover, Petta et al [41] demonstrated that vitamin D serum levels were significantly lower in chronic hepatitis C genotype 1 than in the controls and lower levels were associated with increasing stage of fibrosis and necroinflammatory activity. Moreover, it has been shown that vitamin D protects against oxidative stress and reduces the inflammatory and fibrogenic activity of liver stellate cells. Gibney et al [42] studied vitamin D deficiency in different infectious disorders. Bilharziasis was found not to be associated with vitamin D deficiency. Taken together, this may explain the significant increase of MMP2 in chronic hepatitis B and C compared to bilharziasis and the controls group. In other words, MMP2 increased in chronic hepatitis B and C due to the proposed associated vitamin D deficiency in such patients. On the other hand, MMP2 level in Bilharziasis group was comparable to the controls as this type of infection is not associated with vitamin D deficiency. Worth to mention that the pattern of serum biochemical markers of fibrosis differs according to the etiology of the chronic liver diseases and markers that might be of significance in certain diseases might not be in others.

Except for EGF-R which showed no statistical significant change among the studied groups, TGF-β1, collagen IV, laminin and MMP-2 had the ability, within acceptable performance, to discriminate patients with significant fibrosis (namely F > 2), while only collagen IV and laminin were able to discriminate those with cirrhosis in chronic hepatitis B and C patients. Collagen IV at a cut-off value of 132 ng/ml performed better than the other markers in identifying significant fibrosis and cirrhosis. Murawaki et al found that serum collagen IV can be used as a diagnostic aid for the detection of liver fibrosis at a cut-off value of 110 ng/ml with sensitivity 75% [43].

In conclusion, TGF-β1, collagen IV, laminin and MMP-2 may be used to predict significant fibrosis and/or cirrhosis in children with chronic hepatitis B and C. Among them, collagen IV was the most sensitive and specific. That is to say, non-invasive markers will likely reduce but not replace the need for liver biopsy, which may be useful in monitoring of disease development and treatment effectiveness and might be an inseparable part of assessment of chronic hepatopathies.
